# Sex modulates the ApoE ε4 effect on brain tau deposition measured by ^18^F-AV-1451 PET in individuals with mild cognitive impairment

**DOI:** 10.7150/thno.35366

**Published:** 2019-07-09

**Authors:** Min Liu, Manish D Paranjpe, Xin Zhou, Phan Q. Duy, Manu S Goyal, Tammie L.S. Benzinger, Jie Lu, Rongfu Wang, Yun Zhou

**Affiliations:** 1 Department of Nuclear Medicine, Peking University First Hospital, Beijing, China; 2 The Russell H. Morgan Department of Radiology and Radiological Science, Johns Hopkins University School of Medicine, Baltimore, MD, United States of America; 3 Harvard-MIT Program in Health Sciences and Technology, Harvard Medical School, Boston, MA, United States of America; 4 Department of Neurology, University of Pennsylvania School of Medicine, Philadelphia, PA, United States of America; 5 Medical Scientist Training Program, Yale University School of Medicine, New Haven, CT, United States of America; 6 Mallinckrodt Institute of Radiology, Washington University in St. Louis School of Medicine, St. Louis, MO, United States of America; 7 Department of Radiology, Xuanwu Hospital of Capital Medical University, Beijing, China

**Keywords:** tau deposition, ApoE ε4, mild cognitive impairment, ^18^F-AV-1451, partial volume correction

## Abstract

The strongest genetic risk factor for Alzheimer's disease (AD) is the Apolipoprotein E type 4 allele (ApoE ε4). The interaction between sex and ApoE ε4 carrier status on AD risk remains an area of intense investigation. We hypothesized that sex modulates the relationship between ApoE ε4 carrier status and brain tau deposition (a quantitative endophenotype in AD) in individuals with mild cognitive impairment (MCI).

**Methods**: Preprocessed ^18^F-AV-1451 tau and ^18^F-AV-45 amyloid PET images, T1-weighted structural magnetic resonance imaging (MRI) scans, demographic information, and cerebrospinal fluid (CSF) total tau (t-tau) and phosphorylated tau (p-tau) measurements from 108 MCI subjects in the Alzheimer's Disease Neuroimaging Initiative (ADNI) database were included. After downloading pre-processed images from ADNI, an iterative reblurred Van Cittertiteration partial volume correction (PVC) method was applied to all PET images. MRIs were used for PET spatial normalization. Regions of interest (ROIs) were defined in standard space, and standardized uptake value ratio (SUVR) images relative to cerebellum were computed. ApoE ε4 by sex interaction analyses on ^18^F-AV-1451 and CSF tau (t-tau, p-tau) were assessed using generalized linear models. The association between ^18^F-AV-1451 SUVR and CSF tau (t-tau, p-tau) was assessed.

**Results**: After applying PVC and controlling for age, education level and global cortical ^18^F-AV-45 SUVR, we found that the entorhinal cortex, amygdala, parahippocampal gyrus, posterior cingulate, and occipital ROIs exhibited a significant ApoE ε4 by sex interaction effect (false discovery rate *P* < 0.1) among MCI individuals. We also found a significant ApoE ε4 by sex interaction effect on CSF t-tau and p-tau. ^18^F-AV-1451 SUVR in the 5 ROIs with ApoE ε4 by sex interaction was significantly correlated with CSF p-tau and t-tau.

**Conclusions**: Our findings suggest that women are more susceptible to ApoE ε4-associated accumulation of neurofibrillary tangles in MCI compared to males. Both CSF tau (p-tau, t-tau) and brain tau PET are robust quantitative biomarkers for studying ApoE ε4 by sex effects on brain tau deposition in MCI participants.

## Introduction

Alzheimer's disease (AD) is the leading cause of dementia in the elderly. AD is characterized by the presence of amyloid β (Aβ) plaques and hyperphosphorylated tau neurofibrillary tangles (NFT) which are thought to be responsible for neurodegeneration and subsequent cognitive dysfunction. The lack of therapies for AD highlights the clinical need to better understand the environmental, genetic, and demographic risk factors underlying disease risk and progression. Apolipoprotein E type 4 allele (ApoE ε4) is the largest genetic risk factor for AD development 1, 2. ApoE ε4 carriers exhibit faster cognitive decline 3, 4, increased amyloid β deposition 5, higher NFT density 6 and increased glucose hypometabolism 7 than ApoE ε4 non-carriers. In addition to genetics, sex is an important demographic factor that influences AD risk. Compared to males, females are reported to have higher incidence of AD 8, worse cognitive functions when they have AD 9, and increased AD pathology 10, 11. The interaction between sex and ApoE ε4 carrier status on cognitive decline remains an area of intense investigation. Sex has been shown to modulate the effects of ApoE ε4 in AD clinical conversion risk. Strikingly, healthy older females and females with mild cognitive impairment (MCI) who carry the ApoE ε4 allele are more likely to develop AD than their male counterparts 12, 13. Female ApoE ε4 carriers exhibit higher tau levels in the cerebrospinal fluid (CSF) compared to male carriers 13, especially among amyloid positive females 14.

Neuroimaging shows promise in early AD diagnosis. The novel PET tracer ^18^F-AV-1451 (also called ^18^F-T807, ^18^F-flortaucipir) showed higher selectivity of >25 fold for paired helical filaments of hyperphosphorylated tau (PHF-tau) compared to amyloid β 15. The ^18^F-AV-1451 radiotracer displays favorable pharmacokinetic properties, good overlap with PHF-tau deposits as described by Braak staging 16, and strong association with dementia severity 17. These characteristics make ^18^F-AV-1451 a useful imaging biomarker for studying AD and dementia.

The analysis of ApoE ε4 by sex interaction in tau PET imaging is critical for understanding the role of sex and ApoE ε4 on brain tau PET as a quantitative AD endophenotype. Uncovering regions with ApoE by sex interaction in MCI patients will also help in the development of precision-medicine therapies targeted to sex and ApoE-stratified patient groups in MCI. Further, this analysis will also help to define more appropriate sex and ApoE-stratified imaging-based endpoints for clinical trials in AD. In this study we examined whether sex modulates the ApoE ε4 effect on brain tau deposition measured by ^18^F-AV-1451 PET among MCI subjects in the Alzheimer's Disease Neuroimaging Initiative (ADNI).

## Methods

### Participants

In this cross-sectional study, we collected subjects with available ApoE ε4 genotyping information and ^18^F-AV-1451 PET, ^18^F-AV-45 PET and T1-weighted structural magnetic resonance imaging (MRI) scans from ADNI. 108 MCI subjects were included. For every subject, only his or her most recent ^18^F-AV-1451 PET and matched T1-weighted MRI scan in the same scanning visit were included. The most recent ^18^F-AV-45 PET scan was also included for each subject.

All MCI subjects had a subjective memory concern reported by a clinician, abnormal memory function on the education-adjusted Logical Memory II subscale, and a clinical dementia rating of 0.5 or higher. Further, all MCI subjects were deemed to have cognitive and functional performance that was sufficiently intact to not merit a diagnosis of dementia by the site physician.

A full list of study inclusion/exclusion criteria can be found at https://adni.loni.usc.edu/wp-content/uploads/2008/07/adni2-procedures-manual.pdf.

### ApoE Genotyping, CSF Aβ, t-tau, and p-tau Assessments

Peripheral blood (10 mL) was collected from study participants to be used for ApoE ε4 genotyping. Restriction enzyme isoform genotyping was performed on extracted DNA to test for the presence of the ApoE ε4 genotype, as described previously 18. ApoE ε4 carriers were defined as individuals with at least one ε4 allele (either ε4/ ε4, ε4/ ε3 or ε4/ ε2). Non-carriers were defined as individuals with no ε4 allele.

CSF samples were acquired through lumbar puncture as previously described by ADNI: http://adni.loni.usc.edu/methods/documents/. Concentration of CSF biomarkers including Aβ, t-tau, and p-tau, were assessed using the xMAP Luminex platform (Luminex Corp., Austin, TX) platform. For more details regarding CSF specimen collection and analyte measurement please refer to ADNI.

### MRI and PET Acquisition and Processing

T1-weighted MRI and pre-processed ^18^F-AV-1451 and ^18^F-AV-45 PET images were downloaded from the ADNI database (http://adni.loni.usc.edu/). The PET images had been previously aligned, averaged, reoriented and then interpolated into a standard image and voxel size (image volume 160×160×96, 1.5×1.5×1.5 mm in x, y, z), and smoothed to a uniform resolution of 8 mm in full width at half maximum (FWHM) by the ADNI consortium.

We further processed the downloaded PET images using Statistical Parametric Mapping (SPM12, Wellcome Department of Imaging Neuroscience, London, United Kingdom) and MATLAB (The MathWorks Inc.). Briefly, all PET images were coregistered to matched structural MRI images. The MRI images were normalized to standard Montreal Neurologic Institute (MNI) space using SPM12 and VBM8 toolbox with a MRI template (image volume: 121×145×121, voxel size: 1.5×1.5×15 mm in x, y, z). The transformation parameters determined by MRI spatial normalization were then applied to the coregistered PET images for PET spatial normalization. Regions of interest (ROIs) including cerebellum gray matter for reference tissue were manually drawn on the MRI template using PMOD (PMOD Technologies Ltd., Zürich, Switzerland) in standard MNI space. ROI templates developed previously in the Johns Hopkins Department of Radiology were used in this study 7, 19-21. To measure whole brain amyloid deposition, a global cortex was defined as a union of orbital frontal, prefrontal, superior frontal, lateral temporal, parietal, posterior precuneus, occipital, anterior cingulate, and posterior cingulate. Standard uptake value ratio (SUVR) images were calculated relative to the cerebellum. ROI SUVRs were obtained by calculating mean SUVR within ROIs on the SUVR images in the MNI space.

A partial volume correction (PVC) was applied to the processed ^18^F-AV-1451 and ^18^F-AV-45 PET images to correct or minimize potential underestimation in PET measurement due to low image resolution, especially for small tissues as the amygdala and striatum. In brief, an iterative reblurred Van Cittertiteration method was used for PVC on the mean images, where a 3-D Gaussian kernel of 8 mm FWHM was used for spatial smoothing function h, step length α=1.5, and the iteration was stopped if relative percent change of PVC images < 1% 22.

### Statistical Analysis

To increase statistical power, a ROI feature selection method was used to select regions of interest for ApoE by sex interaction analyses. Specifically, we selected 13 cortical and subcortical regions including the entorhinal cortex, amygdala, fusiform, parahipppocampal gyrus, occipital, lateral temporal, parietal, posterior precuneus, posterior cingulate, orbital frontal cortex, prefrontal cortex, superior frontal, and anterior cingulate, using ROI templates we developed previously in the Johns Hopkins Department of Radiology 7, 19-21. These 13 regions were either previously determined by our group to significantly differ in ^18^F-AV-1451 PET SUVR between cognitively normal, MCI and AD patients 23 or were required to define global cortical amyloid 19. All false discovery rate (FDR) corrections were applied using these 13 ROIs. A depiction of the 13 ROIs used in the study are presented in MNI space in **Figure S1** in the Supplement.

Statistical Analysis System (SAS version 9.4, SAS Institute, Inc) was used for all statistical analyses. Two generalized linear models (GLMs) with and without controlling for global cortical ^18^F-AV-45 SUVR were fit for ApoE ε4 carrier status by sex interaction analyses:

ROI_SUVR (^18^F-AV-1451) ~ Age + Educational level + Global cortex_SUVR (^18^F-AV-45) + Sex:ApoE ε4 status

ROI_SUVR (^18^F-AV-1451) ~ Age + Educational level + Sex:ApoE ε4 status

The GLM models were fit for each ROI SUVR and the ApoE ε4 by sex term was evaluated to identify regions with significant ApoE ε4 by sex interaction effect on ^18^F-AV-1451 uptake. *P* values of ApoE ε4 by sex term were corrected for multiple comparisons using a Benjamini-Hochberg method with FDR < 10% defined as significant, consistent with previous studies 24-26 .

Estimated marginal means (LS-means) of ApoE ε4 carriers and non-carriers stratified by sex were calculated using the interactive models in order to investigate the effects of ApoE ε4 on ^18^F-AV-1451 in women and men separately. Differences in LS-means between ApoE ε4 carriers and non-carriers in males and females separately were evaluated. In addition, we also evaluated differences in LS-means between males and females in ApoE ε4 carrier and non-carrier groups separately. *P* values were corrected for multiple comparisons using the Tukey-Kramer method 27 (maximum experimentwise error rate *P* <0.05).

Similar to the brain tau PET analyses, ApoE ε4 by sex interaction effects on CSF t-tau and p-tau were analyzed by fitting a GLM and controlling for age and education level. We fit additional models controlling for either global cortical ^18^F-AV-45 amyloid SUVR or CSF amyloid.

To investigate whether the regional ^18^F-AV-1451 was significantly associated with AD-relevant biomarkers such as CSF t-tau and p-tau, the correlation between CSF tau (t-tau, p-tau) and regional ^18^F-AV-1451 was analyzed by linear regression.

### Data Availability

All datasets used during the current study are available in the ADNI repository, http://adni.loni.usc.edu/.

## Results

### Cohort Characteristics

A total of 108 ADNI subjects (38 ApoE ε4 carriers and 70 ApoE ε4 non-carriers; 42 women and 66 men) were included in the study. Participant characteristics are summarized in the **Table 1**. The mean±SD age of our study participants was 78±7.4 years. No significant differences in age, MMSE score, and global cortical amyloid SUVR were found between women and men in the ApoE ε4 carrier (two-sample t-test *P* > 0.05) and non-carrier groups (two-sample t-test *P* > 0.05). A moderate difference in education level was found between women and men in the ApoE ε4 carriers (two-sample t-test *P* = 0.01) and non-carriers (two-sample t-test *P* =0.02) groups. Chi-squared analysis revealed no significant differences in the proportion of ApoE ε4ε4/ ε4ε3/ ε4ε2 genotype individuals between men and women (*P* = 0.38). A full list of demographic variables with statistics are listed in **Table 1**.

### ApoE ε4 by sex interaction on ^18^F-AV-1451 ROI SUVR

ROIs with significant ApoE ε4 by sex interaction on ^18^F-AV-1451 SUVR were identified with and without controlling for global cortical amyloid (**Table 2**) using the 13 ROIs described in the Methods. In MCI individuals, we found the entorhinal cortex, amygdala, parahippocampal gyrus, posterior cingulate, and occipital ROIs to exhibit a significant ApoE ε4 carrier status by sex effect (FDR* P* < 0.1) after applying PVC and controlling for global cortical amyloid uptake measured by ^18^F-AV-45 PET. Notably, only the entorhinal cortex reached significance (FDR *P* < 0.1) in the non-PVC data (**Table 2**). For the GLM without controlling for global cortical amyloid uptake, we found the entorhinal cortex, amygdala, fusiform, parahippocampal gyrus, posterior cingulate, and occipital ROIs to exhibit a significant ApoE ε4 by sex effect (FDR* P* < 0.1; **Table 2**) in both PVC and non-PVC data.

**Figure 1:**
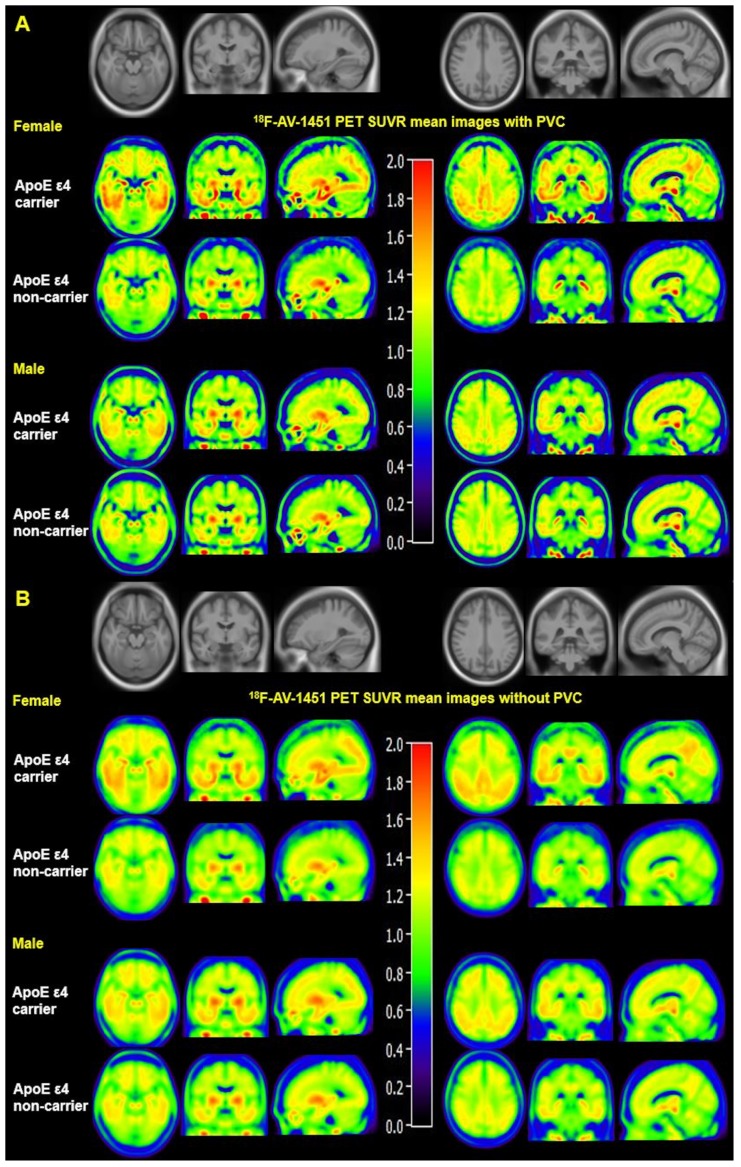
** Mean ^18^F-AV-1451 PET SUVR images with and without partial volume correction in MCI individuals.** Mean images were generated by computing the mean of images from ApoE ε4 carriers and non-carriers separately. Partial volume corrected images (A) show increased contrast and spatial resolution compared to non-PVC images (B). Both PVC and non-PVC images show an interaction effect between sex and ApoE ε4 status. Note that the mean images are averaged over all participants in each sex-ApoE ε4 group.

**Figure 2:**
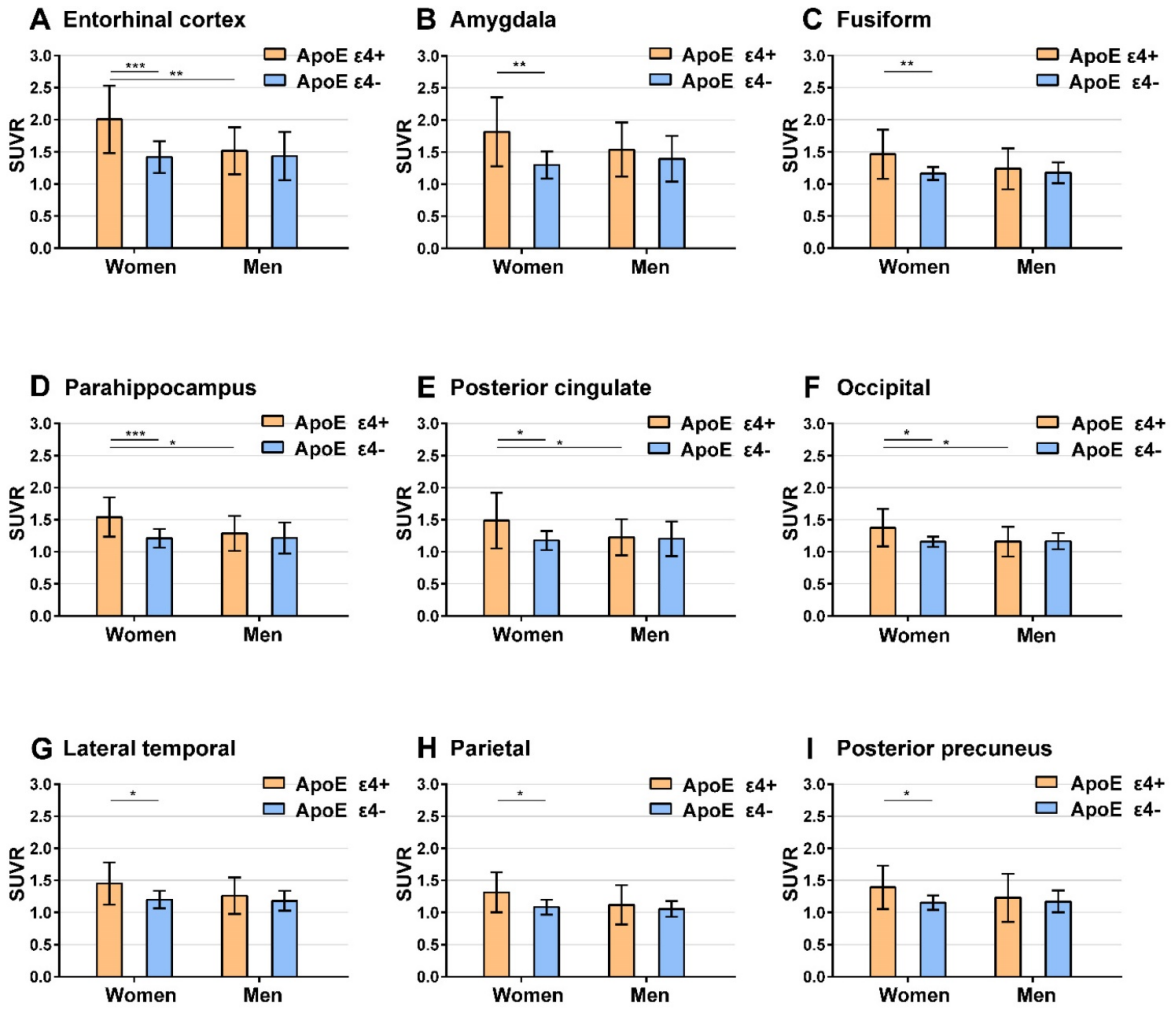
** Regions with significant ApoE ε4 effect on ^18^F-AV-1451 SUVR.** Bar graphs showing ROIs SUVR (mean with error bars depicting SD) of tau ^18^F-AV-1451 PET imaging between ApoE ε4 carriers and non-carriers in regions with significant ApoE ε4 effect. *P* value were defined using a two-sample t-test to compare SUVR between ApoE ε4 carriers and ApoE ε4 non-carriers in male and female subgroups, and between males and females in ApoE ε4 carrier and ApoE ε4 non-carrier subgroups. *** *P* < 0.001; ** *P* < 0.01; * *P* < 0.05. Note: Short significance lines indicate comparison between ApoE ε4 carriers and non-carriers using a two-sample t-test. Long significance lines indicate comparison between men and women using a two-sample t-test.

Mean SUVR images with and without PVC from MCI females and males are displayed in **Figure 1**. **Figure 1A** visually suggests that PVC increases ^18^F-AV-1451 SUVR contrast between ApoE ε4 carriers and non-carriers in the entorhinal cortex, amygdala, fusiform, parahippocampal gyrus, posterior cingulate, and occipital ROIs compared to non-PVC mean SUVR images (**Figure 1B**). **Figure 1** also suggests that presence of the ApoE ε4 allele is associated with more tau deposition in women compared to men. Quantified SUVR from ^18^F-AV-1451 images with PVC in ROIs stratified by sex and ApoE status are displayed in **Figure 2**.

Upon comparing SUVR from ApoE ε4 carriers with ApoE ε4 non-carriers, we found a significant ApoE ε4 effect in the entorhinal cortex, amygdala, fusiform, parahippocampus, posterior cingulate, occipital cortex, lateral temporal, parietal, and posterior precuneus among women (two-sample t-test, *P* < 0.05). In contrast, no ROIs exhibit a significant ApoE ε4 effect in men.

### ApoE ε4 and sex-stratified analysis

Given that we saw significant ApoE ε4 by sex interaction effects in MCI patients, we analyzed the effect of ApoE ε4 on ^18^F-AV-1451 stratified by sex (**Table 3**) after adjusting for education, age and amyloid. Specifically, we analyzed the difference in marginal means between ApoE ε4 carriers and non-carriers stratified by sex. All 5 ROIs (entorhinal cortex, amygdala, parahippocampal gyrus, posterior cingulate and occipital ROIs) with significant ApoE ε4 by sex interaction effect displayed a significant ApoE ε4 effect in women when the marginal means were not adjusted for global cortical ^18^F-AV-45 SUVR. The entorhinal cortex, amygdala, parahippocampal gyrus, and posterior cingulate retained a significant ApoE ε4 effect in women when the marginal means were adjusted for global cortical ^18^F-AV-45 SUVR (FDR < 0.1). No ROIs exhibit a significant ApoE ε4 effect in men.

We also analyzed the difference in marginal means between males and females stratified by ApoE ε4 carrier status (**Table 3**). Four out of the 5 ROIs (entorhinal cortex, parahippocampal gyrus, posterior cingulate and occipital ROIs) with significant ApoE ε4 by sex interaction showed a significant sex effect in ApoE ε4 carriers with or without adjusting marginal means for global cortical ^18^F-AV-45 SUVR. These regions retained a significant sex effect in ApoE ε4 carriers when adjusted for global cortical ^18^F-AV-45 SUVR. In contrast, no ROIs exhibit a significant sex effect in ApoE ε4 non-carriers. Non-PVC based results are presented in **Table S1** in the Supplement.

### Association between regional ^18^F-AV1451 and CSF p-tau and t-tau

**Figure 3:**
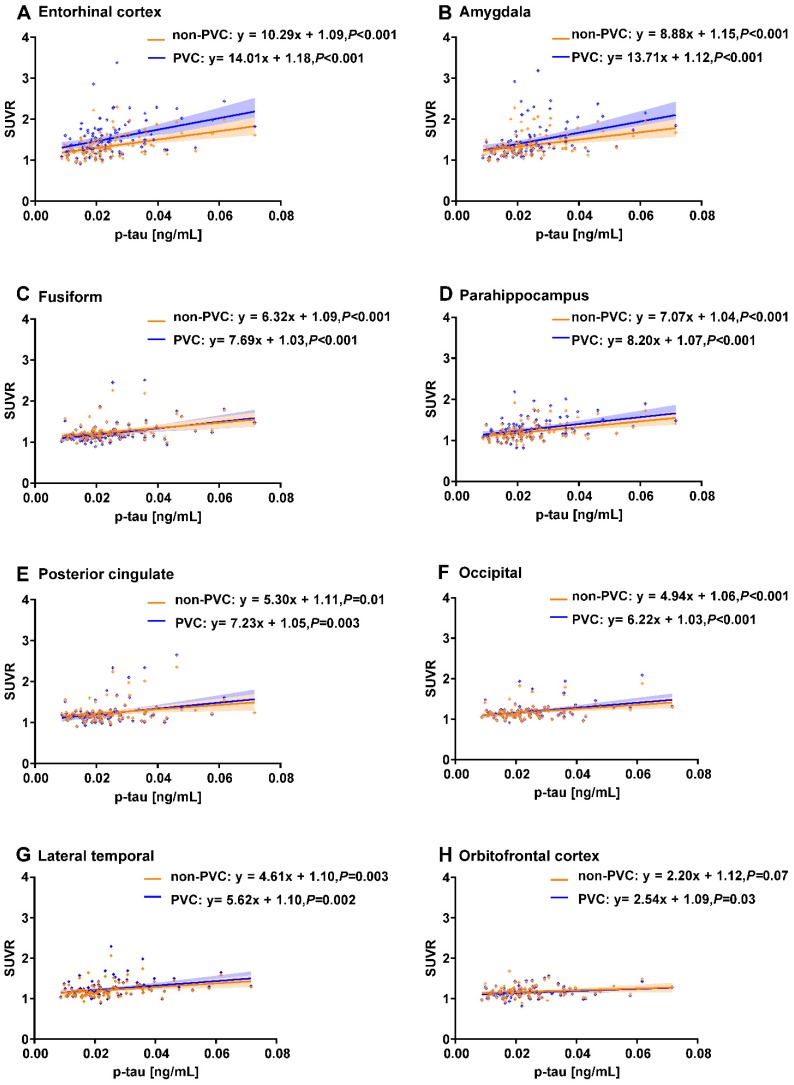
** Correlations between regional ^18^F-AV-1451 SUVR and CSF p-tau measurements.** Line graphs showing correlation between CSF p-tau (ng/mL) and ^18^F-AV-1451 SUVR. Both non-PVC and PVC PET data are shown for ROIs in the entorhinal cortex (A), amygdala (B), fusiform (C), parahippocampus gyrus (D), posterior cingulate (E), occipital (F), lateral temporal (G), and orbitofrontal cortex (H). Fitted lines, *P*-values, and 95% confidence intervals are displayed from linear regression models.

We identified 97 ADNI subjects with CSF t-tau and p-tau measurements. We observed that CSF p-tau was significantly associated with the PVC based ^18^F-AV-1451 SUVR in the entorhinal cortex, amygdala, fusiform, parahippocampal gyrus, posterior cingulate, occipital, lateral temporal, and orbitofrontal cortex (*P* < 0.05; **Figure 3**). The orbitofrontal cortex failed to reach significance in the non-PVC data (*P* = 0.07). CSF t-tau was also significantly associated with ^18^F-AV-1451 PVC PET in all 8 of these regions. The posterior cingulate (*P* = 0.08) and orbitofrontal cortex (*P* = 0.07) failed to reach significance in the non-PVC data.

**Figure 4:**
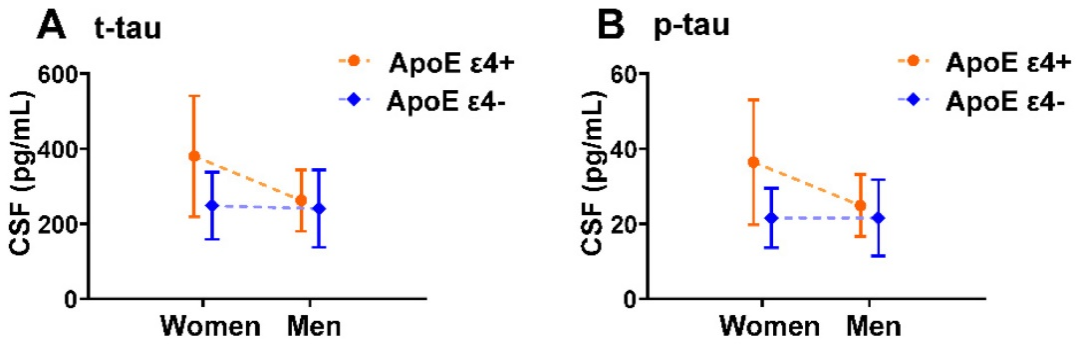
** ApoE ε4 by sex interaction effects on CSF t-tau, p-tau in MCI individuals.** Plots of mean with error bar (standard deviation) showing CSF t-tau (A) and p-tau (B) in each sex-ApoE ε4 subgroup. Dashed lines indicate the direction of the ApoE ε4 effect. This graphical depiction of ApoE ε4 by sex interaction effects on CSF t-tau and p-tau is supported by statistical results in Table 4.

We also analyzed potential ApoE ε4 by sex interaction effects in CSF t-tau and p-tau. We found a significant ApoE ε4 by sex interaction effect on CSF t-tau and p-tau with and without controlling for amyloid in the 97 ADNI subjects with CSF t-tau and p-tau measurements (**Table 4**). Mean CSF t-tau and p-tau levels stratified by sex and ApoE ε4 carrier status are displayed graphically in **Figure 4**.

## Discussion and Conclusion

This cross-sectional analysis provides evidence of a significant ApoE ε4 carrier status by sex interaction effect on brain tau measured using ^18^F-AV-1451 PET in MCI patients. Using 108 ADNI participants, we observed a significant ApoE ε4 by sex interaction effect (FDR *P* < 0.1) on ^18^F-AV-1451 tau binding in the entorhinal cortex, amygdala, parahippocampal gyrus, posterior cingulate, and occipital cortex among MCI individuals. Further, our ROI based results suggest that PVC improved PET spatial resolution and contrast in this study. Specifically, we observe a near but not significant ApoE ε4 by sex interaction effect in AV-1451 binding without PVC (entorhinal cortex was significant without PVC) (**Table 2**). Also, as a result of applying PVC, we are confident that changes in ^18^F-AV-1451 reported here are genuine metabolic changes associated with ApoE ε4 rather than due to volume-related or technical biases.

A recently published study analyzed ApoE ε4 by sex interaction effects on ^18^F-AV-1451 PET using healthy elderly individuals 28. The authors found a significant ApoE ε4 by sex interaction effect in a meta-ROI containing the entorhinal cortex, inferior temporal cortex, amygdala, fusiform gyrus, and parahippocampal cortex, but no significant ApoE ε4 by sex interaction effect on the entorhinal cortex alone. In contrast to this prior study that investigated only cognitively normal individuals, our study focused on a cohort with MCI demonstrating that this ApoE ε4 by sex interaction on tauopathy persists in individuals progressing towards symptomatic AD. This result is particularly important when considering clinical trials aimed at an MCI cohort. Furthermore, our study identifies this interaction using different imaging processing methods and additional ROIs, suggesting that the ApoE ε4 by sex interaction on tauopathy identified by both studies is robust to different methodologies within the ADNI cohort.

Importantly, in the present study we controlled for global cortical amyloid load measured by ^18^F-AV-45 PET. Recent work shows that amyloid might increase brain tau through mechanisms involving Sirt3 29, GSK3β 30, 31 or Cdk5 32. In imaging studies involving brain tau, it is important to control for the potential confounding effects of amyloid on tau. In our data, we observe a significant ApoE ε4 by sex interaction on AV-1451 PET after controlling for global cortical amyloid burden, suggesting that the ApoE ε4 allele exerts a sex-dependent effect on brain tau, independent of amyloidosis. These results are supported by recent biochemical studies demonstrating that ApoE ε4 triggers higher neuronal levels of phospho-tau independent of amyloid 33. Further, transgenic mice overexpressing ApoE ε4 exhibit greater hippocampal phospho-tau and exhibit deficits in tau clearance independent of amyloid pathology 34. It should be noted however that in spite of possible amyloid-independent mechanisms, additional statistical models analyzing amyloid by sex interaction effects suggest that our originally-reported ApoE by sex interaction effect on brain tau in the occipital cortex may be partly due to amyloid as females may be more susceptible to amyloid-induced tauopathy in this region (**Result S1** in the Supplement).

Our ApoE ε4 and sex-stratified analysis help explain the nature of ApoE ε4 by sex interaction effect in 4 out of the 5 ROIs with a significant ApoE ε4 by sex interaction effect (entorhinal cortex, parahippocampal gyrus, posterior cingulate and occipital ROIs). In these ROIs, there is no sex difference in ^18^F-AV-1451 among ApoE ε4 non-carriers. However, in ApoE ε4 carriers, we observed a significant sex effect (**Table 3**). This suggests that the presence of ApoE ε4 allele is associated with significantly higher levels of brain tau deposition in females as compared to males.

As a further measure of clinical relevance of our study, we found that ^18^F-AV-1451 signal in all 5 ROIs with significant ApoE ε4 by sex interaction effect is also associated with CSF p-tau (**Figure 3**). Previous studies have found an ApoE ε4 by sex interaction effect on CSF p-tau, a well-studied biomarker in AD 13, 14. Our data adds to the clinical validity of CSF p-tau by elucidating a brain-based correlate. Overall, by integrating ^18^F-AV-1451 PET and CSF p-tau data, we find a robust relationship between brain tau and biomarker changes outside the brain.

A possible additional explanation for the sex differences identified in our study might be that women could be cognitively more resilient to the effects of tauopathy. Recent findings suggest that women may have on average “younger brain predicted age” based on structural brain MRI 35 and metabolic brain PET imaging 36. Similarly, younger individuals with autosomal dominant AD show markedly increased tauopathy compared to late onset AD individuals despite similar degrees of cognitive impairment 37. It is possible then that due to increased brain resilience, women with more ApoE ε4 mediated tauopathy than men can nevertheless remain cognitively normal as demonstrated by Buckley et al. 28 or mildly impaired as demonstrated in our results. In order to further test the hypothesis that women may be more resilient to higher loads of tauopathy than men in MCI, we refitted our ApoE ε4 by sex interaction models controlling for MMSE score (**Table S2** in the Supplement). These results show that all five regions with significant ApoE ε4 by sex interaction effect in MCI in the original model (**Table 2**) are also significant when controlling for MMSE score. This analysis further suggests that compared to men, women experience greater ApoE ε4-mediated tauopathy while remaining at the same level of cognitive deficit. Notably the idea that sex differences in brain resilience to AD might help explain our findings is not mutually exclusive from the idea that sex also influences the risk to AD pathology; both sex brain differences and ApoE status might carry both risks and resilience to neurodegeneration and dementia, as well as vascular disease. Further studies to disentangle these potentially complex relationships are needed.

This study has clinical implications for precision medicine. Our results demonstrate that female ApoE ε4 carriers exhibit greater tau accumulation than their male counterparts. Several anti-tau clinical trials are underway 38, 39. In designing clinical trials, our results suggest that the dosage of anti-tau antibodies should be modified by ApoE ε4-sex group. In future efforts to validate tau as a quantitative endophenotype or clinical outcome measure, studies should stratify patient cohorts by ApoE ε4-sex group. Likewise, in mechanistic studies exploring tau-dependent mechanisms underlying ApoE ε4-mediated AD risk, results should be analyzed separately in males and females.

As a limitation to our study, it should be noted that our study contained a small percentage (35%) of ApoE ε4 carriers. While this proportion is in line with previously published studies and the larger ADNI cohort 40, 41, possible selection biases may be present in our cohort. Future studies should be conducted in larger cohorts to minimize selection biases. Our study is however balanced in terms of proportion of female and male ApoE ε4 carriers (females: 36% ApoE ε4 carriers vs males: 35% ApoE ε4 carriers;* P* = 0.93, Chi-Square test).

## Supplementary Material

Supplementary Result S1, Tables S1-2, and Figure S1.

## Figures and Tables

**Table 1:** Study cohort characteristics

	Mean±SD [Range]				*P* value^a^			
Characteristic	Women ApoE ε4+ (n=15)	Women ApoE ε4 - (n=27)	Men ApoE ε4+ (n=23)	Men ApoE ε4 - (n=43)	Women	Men	ApoE ε4+	ApoE ε4 -
					ε4+ vs ε4 -	ε4+ vs ε4 -	Women vs Men	Women vs Men
Age, year	74.4±6.8 [61.2-84.5]	79.3±6.4 [68.1-92.6]	76.7±8.4 [59.6-90.3]	79.8±7.1 [67.8-93.2]	0.03	0.12	0.39	0.78
Education, years	14.1±2.7 [8.0-20.0]	15.4±2.5 [12.0-19.0]	16.8±3.2 [12.0-20.0]	17.0±2.9 [8.0-20.0]	0.12	0.76	0.01	0.02
White race, No (%)	15(100)	24(89)	22(96)	43(100)	0.18	0.17	0.41	0.03
MMSE Score	26.8±2.4 [23.0-30.0]	28.0±2.4 [19.0-30.0]	27.4±2.6 [22.0-30.0]	28.1±2.0 [23.0-30.0]	0.13	0.22	0.45	0.80
global cortical amyloid SUVR	1.56±0.35 [1.02-2.35]	1.20±0.21 [0.96-1.69]	1.47±0.35 [0.99-2.20]	1.28±0.40 [0.91-3.12]	0.001	0.06	0.43	0.28
CSF Aβ-42 (pg/mL)	933.0±398.1 [469.0-1700]	1326.6±425.4 [426-1700]	845.3±435.0 [248.1-1700]	1272.3±415.6 [396.6-1700]	0.01	<0.001	0.55	0.62
CSF t-tau (pg/mL)	380.5±160.8 [234.6-750.0]	248.5±89.2 [100.2-443.5]	262.7±82.4 [115.4-426.4]	240.7±103.2 [121.4-553.5]	0.01	0.41	0.03	0.75
CSF p-tau (pg/mL)	36.5±16.6 [20.6-71.6]	21.6±7.9 [8.7-38.6]	24.9±8.3 [10.5-42.3]	21.6±10.2 [9.9-52.3]	0.01	0.22	0.03	1
Interval between ^18^F-AV-1451 and ^18^F-AV-45, month	5.5±7.3 [0.0-27.0]	4.3±5.1 [0.0-16.0]	3.78±4.51 [0.0-13.0]	3.30±4.85 [0.0-17.0]	0.54	0.70	0.37	0.40
ε4 ε4/ ε4 ε3/ ε4 ε2 in ApoE ε4 carriers	3/11/1		7/16/0				0.38	

^a^ *P* value was defined using a two-sample t test to compare age, education level, MMSE Score, global cortical amyloid SUVR, CSF biomarkers and AV1451-AV45 scanning interval between ApoE ε4 carriers and ApoE ε4 non-carriers in male and female subgroups, and between males and females in ApoE ε4 carrier and ApoE ε4 non-carrier subgroups. A Chi-square test was used to compare differences in proportion of Caucasian participants between ApoE ε4 carriers and ApoE ε4 non-carriers in male and female subgroups, and between males and females in ApoE ε4 carrier and ApoE ε4 non-carrier subgroups. A Chi-squared test was used to compare proportion of ε4ε4/ ε4ε3/ ε4ε2 genotype individuals between males and females in the ApoE ε4 carrier group.

**Table 2:** ApoE ε4 carrier status by sex interaction effect in MCI participants

		PVC			non-PVC		
Model	Characteristic	Standardized β(95%CI) ^a^	ApoE ε4 x sex *P* value ^b^	ApoE ε4 x sex Adjusted *P* value^c^	Standardized β(95%CI) ^a^	ApoE ε4 x sex *P* value ^b^	ApoE ε4 x sex Adjusted *P* value^c^
Adjusted for global cortical amyloid level	Entorhinal Cortex	0.39(0.14-0.64)	0.003	0.03	0.34(0.10-0.59)	0.01	0.09
	Amygdala	0.30(0.03-0.55)	0.03	0.07	0.26(0.00-0.52)	0.05	0.16
	Fusiform	0.25(0.00-0.50)	0.05	0.11	0.21(-0.04-0.46)	0.10	0.22
	Parahippocampal	0.29(0.03-0.54)	0.03	0.07	0.26(0.01-0.51)	0.04	0.16
	Posterior Cingulate	0.31(0.04-0.57)	0.03	0.07	0.25(-0.02-0.51)	0.07	0.18
	Occipital	0.34(0.09-0.58)	0.01	0.06	0.30(0.05-0.55)	0.02	0.11
	Orbital Frontal	0.16(-0.12-0.45)	0.25	0.33	0.06(-0.23-0.340	0.68	0.74
	Prefrontal	0.07(-0.2-0.35)	0.61	0.66	0.03(-0.25-0.31)	0.83	0.83
	Superior Frontal	0.01(-0.26-0.28)	0.96	0.96	-0.07(-0.34-0.20)	0.59	0.70
	Lateral Temporal	0.18(-0.07-0.43)	0.15	0.22	0.15(-0.10-0.40)	0.24	0.40
	Parietal	0.18(-0.07-0.45)	0.15	0.22	0.13(-0.13-0.39)	0.33	0.44
	Posterior Precuneus	0.21(-0.07-0.48)	0.14	0.22	0.16(-0.11-0.43)	0.24.	0.40
	Anterior Cingulate	-0.11(-0.38-0.16)	0.42	0.50	-0.13(-0.41-0.14)	0.34	0.44
Not adjusted for global cortical amyloid level	Entorhinal Cortex	0.43(0.18-0.69)	0.001	0.01	0.40(0.14-0.65)	0.003	0.04
	Amygdala	0.34(0.07-0.60)	0.01	0.03	0.30(0.04-0.56)	0.03	0.09
	Fusiform	0.31(0.05-0.58)	0.02	0.05	0.28(0.00-0.55)	0.04	0.10
	Parahippocampal	0.35(0.08-0.61)	0.01	0.03	0.32(0.06-0.59)	0.02	0.08
	Posterior Cingulate	0.35(0.08-0.63)	0.01	0.03	0.30(0.02-0.57)	0.04	0.09
	Occipital	0.40(0.14-0.67)	0.003	0.02	0.37(0.10-0.64)	0.01	0.04
	Orbital Frontal	0.19(-0.09-0.47)	0.19	0.25	0.09(-0.20-0.37)	0.55	0.65
	Prefrontal	0.10(-0.18-0.39)	0.46	0.53	0.07(-0.22-0.35)	0.64	0.69
	Superior Frontal	0.06(-0.22-0.34)	0.67	0.67	-0.01(-0.30-0.27)	0.93	0.93
	Lateral Temporal	0.26(-0.01-0.53)	0.06	0.12	0.22(-0.05-0.49)	0.11	0.20
	Parietal	0.24(-0.03-0.51)	0.08	0.12	0.19(-0.09-0.46)	0.18	0.26
	Posterior Precuneus	0.25(-0.03-0.52)	0.08	0.12	0.21(-0.07-0.48)	0.15	0.24
	Anterior Cingulate	-0.09(-0.36-0.17)	0.49	0.53	-0.11(-0.39-0.16)	0.43	0.56

^a^ β value is coefficient of ApoE ε4 by sex interaction, 95% CI represents the 95% confidence interval of the ApoE ε4 by sex coefficient. ^b^ *P* value as defined using a generalized linear model to detect significant ApoE ε4 by sex interaction effect in MCI subjects. Age and education were included as covariates. Global cortical amyloid SUVR was also included as a covariate in the upper results. ^c^ Adjusted *P* value as defined using Benjamini-Hochberg procedure to control FDR.

**Table 3:** Effects of ApoE ε4 on ^18^F-AV-1451 stratified by sex with PVC

		LS-mean^a^				*P* value^b^			
Model	Region	Women ApoE ε4+	Women ApoE ε4 -	Men ApoE ε4+	Men ApoE ε4 -	Women	Men	ApoE ε4+	ApoE ε4 -
						ε4+ vs ε4 -	ε4+ vs ε4 -	Women vs Men	Women vs Men
Adjusted for global cortical amyloid level	Entorhinal Cortex	1.96	1.46	1.48	1.44	<0.001	0.97	0.001	1
	Amygdala	1.79	1.33	1.52	1.40	0.003	0.60	0.13	0.88
	Fusiform	1.39	1.20	1.20	1.20	0.08	1	0.08	1
	Parahippocampal	1.48	1.25	1.26	1.23	0.02	0.98	0.03	1
	Posterior Cingulate	1.46	1.21	1.20	1.21	0.05	1	0.04	1
	Occipital	1.31	1.18	1.14	1.19	0.11	0.63	0.01	1
	Orbital Frontal	1.23	1.17	1.13	1.14	0.61	1	0.14	0.70
	Prefrontal	1.16	1.14	1.09	1.10	0.98	0.99	0.37	0.51
	Superior Frontal	1.17	1.14	1.10	1.08	0.98	0.97	0.69	0.49
	lateral temporal	1.38	1.24	1.23	1,21	0.19	0.98	0.12	0.91
	Parietal	1.25	1.11	1.10	1.08	0.21	0.99	0.13	0.91
	Posterior Precuneus	1.35	1.17	1.21	1.18	0.17	0.98	0.34	1
	Anterior Cingulate	0.94	0.97	0.89	0.88	0.88	0.99	0.75	0.04
Not adjusted for global cortical amyloid level	Entorhinal Cortex	2.04	1.41	1.53	1.42	<0.001	0.69	<0.001	1
	Amygdala	1.85	1.29	1.55	1.38	<0.001	0.28	0.10	0.78
	Fusiform	1.45	1.16	1.24	1.18	0.002	0.75	0.06	0.99
	Parahippocampal	1.54	1.21	1.29	1.22	<0.001	0.63	0.02	1
	Posterior Cingulate	1.51	1.18	1.23	1.19	0.003	0.96	0.02	1
	Occipital	1.36	1.15	1.17	1.17	0.003	1	0.01	0.95
	Orbital Frontal	1.24	1.16	1.14	1.13	0.31	1	0.11	0.77
	Prefrontal	1.18	1.13	1.10	1.09	0.73	1	0.30	0.63
	Superior Frontal	1.20	1.12	1.13	1.07	0.49	0.67	0.57	0.71
	Lateral Temporal	1.44	1.20	1.26	1.19	0.01	0.53	0.09	1
	Parietal	1.29	1.08	1.12	1.06	0.01	0.69	0.09	1
	Posterior Precuneus	1.39	1.15	1.23	1.17	0.03	0.79	0.26	0.99
	Anterior Cingulate	0.95	0.97	0.89	0.88	0.97	0.95	0.70	0.05

^a^ LS-mean indicates the least squares (marginal) means in each ApoE ε4-sex groups after adjusting for age and education. Global cortical amyloid SUVR was also included as a covariate in the upper results. ^b^ All *P* values correspond to pairwise LS-mean differences after correcting for multiple comparisons using the Tukey-Kramer method.

**Table 4:** ApoE ε4 by sex interaction effect on CSF t-tau and p-tau in MCI participants

	CSF t-tau		CSF p-tau	
Model	Standardized β(95%CI) ^a^	ApoE ε4 x sex *P* value ^b^	Standardized β(95%CI) ^a^	ApoE ε4 x sex *P* value ^b^
Not adjusted for global cortical ^18^F-AV-45	0.35(0.06-0.62)	0.02	0.36(0.08-0.63)	0.01
Adjusted for CSF amyloid level	0.30(0.03-0.55)	0.03	0.26(0.00-0.52)	0.05
Adjusted for global cortical ^18^F-AV-45 (non-PVC)	0.33(0.04-0.60)	0.02	0.33(0.05-0.60)	0.02
Adjusted for global cortical ^18^F-AV-45 (PVC)	0.33(0.04-0.60)	0.03	0.33(0.05-0.59)	0.02

^a^ β value is coefficient of ApoE ε4 by sex interaction, 95% CI represents the 95% confidence interval of the ApoE ε4 by sex coefficient. ^b^ *P* value as defined using a generalized linear model to detect significant ApoE ε4 by sex interaction effect in MCI subjects. Age and education were included as covariates in each model. Additional covariates of CSF amyloid or global cortical AV45 were added were added as indicated.

## Abbreviations

Aβ: amyloid β
AD: alzheimer's disease
ADNI: alzheimer's disease neuroimaging initiative
ApoE ε4: apolipoprotein E type 4 allele
CSF: cerebrospinal fluid
FDR: false discovery rate
FWHM: full width at half maximum
GLM: generalized linear model
MCI: mild cognitive impairment
MMSE: mini mental state examination
MNI: montreal neurological institute
MRI: magnetic resonance imaging
NFT: neurofibrillary tangles
PET: positron emission tomographic
PHF-tau: paired helical filaments of tau
p-tau: phosphorylated tau
PVC: partial volume correction
ROIs: regions of interest
SAS: statistical analysis system
SPM: statistical parametric mapping
SUVR: standardized uptake value ratio
t-tau: total tau
