# Supplementary material for: Sex modulates the ApoE ε4 effect on brain tau deposition measured by 18F-AV-1451 PET in individuals with mild cognitive impairment
Source: Theranostics. 2019 Jul 9;9(17):4959–70. doi: 10.7150/thno.35366 (PMC6691387; doi:10.7150/thno.35366)
Supplement: Supplementary file 1 — Supplementary Result S1, Tables S1-2, and Figure S1. [file thnov09p4959s1.pdf]

Supplementary Materials

I. Result S1

Additional statistical models to investigate possible confounding effects of amyloid

II. Table S1

ApoE ε4 effects stratified by sex on ^{18}F-AV-1451 PET images without PVC

III. Table S2

ApoE ε4 carrier status by sex interaction effect in MCI participants after controlling for MMSE score

IV. Figure S1

Regions of interest defined in standard Montreal Neurological Institute space

Result S1. Additional statistical models to investigate possible confounding effects of amyloid

Due to the nonlinear distribution of brain amyloid, correcting for amyloid using statistical models may be inaccurate. To further investigate possible confounding effects of amyloid, we fit the following two additional models below. We first fit the model 1:

SUVR(tau) ~ ApoE ε4 + Age + Education + Global cortex_SUVR (^{18}F-AV-45):sex

We found no significant amyloid:sex interaction effects on ^{18}F-AV-1451 tau burden except in the occipital cortex (P =0.001). To assess the effects of ApoE:sex interaction on regional amyloid in our 13 study ROIs, we fit the model 2:

SUVR(amyloid) ~ Age + Education + ApoE ε4:sex

No regions showed a significant ApoE ε4 by sex interaction effect or sex main effect in this model. All 13 ROIs showed a significant ApoE ε4 main effect (P < 0.05). Model 2 indicates that the ApoE ε4 allele is associated with similar degrees of amyloid deposition in males and females. However, model 1 indicate that males and females differ in their association between amyloid and tau deposition (amyloid-induced tau phosphorylation) in the occipital cortex. Taken together, these analyses suggests that our originally-reported ApoE:sex interaction effect on brain tau in the occipital cortex may be partly due to amyloid as females may be more susceptible to amyloid-induced tauopathy in this region.

Table S1. ApoE ε4 effects stratified by sex on 18F-AV-1451 PET images without PVC

		LS-mean[a]				*P* value[b]			
**Model**	**Region**	**Women -ApoE ε4+**	**Women -ApoE ε4 -**	**Men-ApoE ε4+**	**Men-ApoE ε4 -**	**Women ε4+ vs ε4 -**	**Men ε4+ vs ε4 -**	**ApoE ε4+ Women vs Men**	**ApoE ε4 - Women vs Men**
Adjusted for global cortical amyloid level	Entorhinal Cortex	1.61	1.28	1.35	1.29	<0.001	0.75	0.01	1
	Amygdala	1.58	1.28	1.43	1.33	0.003	0.38	0.28	0.85
	Fusiform	1.39	1.22	1.26	1.22	0.06	0.89	0.18	1
	Parahippocampal	1.38	1.18	1.22	1.18	0.01	0.81	0.06	1
	Posterior Cingulate	1.42	1.22	1.25	1.22	0.06	0.96	0.11	1
	Occipital	1.29	1.17	1.15	1.17	0.05	0.98	0.02	1
	Orbital Frontal	1.24	1.19	1.18	1.15	0.65	0.81	0.53	0.63
	Prefrontal	1.18	1.14	1.11	1.09	0.91	0.95	0.52	0.42
	Superior Frontal	1.14	1.12	1.12	1.07	0.99	0.60	0.97	0.44
	lateral temporal	1.33	1.21	1.22	1.18	0.17	0.82	0.20	0.87
	Parietal	1.22	1.09	1.12	1.06	0.19	0.69	0.40	0.95
	Posterior Precuneus	1.36	1.19	1.26	1.21	0.15	0.76	0.61	0.99
	Anterior Cingulate	1.03	1.05	1.00	0.97	0.96	0.83	0.95	0.10
Not adjusted for global cortical amyloid level	Entorhinal Cortex	1.67	1.24	1.39	1.28	<0.001	0.34	0.01	0.95
	Amygdala	1.62	1.25	1.45	1.31	<0.001	0.14	0.22	0.73
	Fusiform	1.44	1.19	1.29	1.21	0.002	0.40	0.13	0.99
	Parahippocampal	1.43	1.15	1.25	1.17	<0.001	0.36	0.04	0.99
	Posterior Cingulate	1.47	1.19	1.27	1.20	0.003	0.67	0.08	1
	Occipital	1.34	1.14	1.18	1.16	0.001	0.94	0.02	0.97
	Orbital Frontal	1.26	1.18	1.19	1.15	0.33	0.61	0.46	0.72
	Prefrontal	1.20	1.13	1.12	1.09	0.52	0.75	0.43	0.56
	Superior Frontal	1.17	1.10	1.14	1.06	0.49	0.22	0.90	0.69
	Lateral Temporal	1.39	1.18	1.25	1.17	0.01	0.30	0.15	0.99
	Parietal	1.26	1.06	1.14	1.05	0.01	0.28	0.30	1
	Posterior Precuneus	1.40	1.17	1.29	1.19	0.02	0.43	0.50	0.96
	Anterior Cingulate	1.04	1.04	1.01	0.96	1	0.65	0.92	0.13

[a] LS-mean indicates least squares (marginal) means in sex- ApoE ε4 subgroups after adjusting for age, education and amyloid.

[b] *P* value was defined after applying a multiple comparison correction (Tukey-Kramer method) for the pairwise differences in LS-means.

Table S2. ApoE ε4 carrier status by sex interaction effect in MCI participants after controlling for MMSE score

		PVC			non-PVC		
**Model**	**Characteristic**	**Standardized β(95%CI) [a]**	**ApoE ε4 x sex *P* value [b]**	**ApoE ε4 x sex Adjusted *P* value[c]**	**Standardized β(95%CI) [a]**	**ApoE ε4 x sex *P* value [b]**	**ApoE ε4 x sex Adjusted *P* value[c]**
Adjusted for global cortical amyloid level	Entorhinal Cortex	0.37（0.14-0.60）	0.002	0.03	0.32(0.09-0.55)	0.01	0.08
	Amygdala	0.27（0.04-0.49）	0.02	0.08	0.23(0.00-0.46)	0.05	0.16
	Fusiform	0.24（-0.01-0.50）	0.06	0.12	0.20(-0.05-0.45)	0.11	0.24
	Parahippocampal	0.27（0.03-0.51）	0.03	0.08	0.25(0.01-0.48)	0.04	0.16
	Posterior Cingulate	0.30（0.03-0.56）	0.03	0.08	0.24(-0.03-0.50)	0.08	0.21
	Occipital	0.33（0.08-0.58）	0.01	0.06	0.30(0.05-0.54)	0.02	0.12
	Orbital Frontal	0.16（-0.13-0.44）	0.27	0.35	0.05(-0.23-0.34)	0.72	0.78
	Prefrontal	0.07（-0.21-0.35）	0.63	0.68	0.02(-0.26-0.31)	0.87	0.87
	Superior Frontal	-0.002（-0.27-0.27）	1	1	-0.08(-0.35-0.19)	0.55	0.65
	Lateral Temporal	0.18（-0.07-0.42）	0.16	0.25	0.14(-0.11-0.39)	0.26	0.44
	Parietal	0.18（-0.08-0.43）	0.17	0.25	0.12(-0.14-0.38)	0.36	0.47
	Posterior Precuneus	0.20（-0.08-0.47）	0.16	0.25	0.15(-0.12-0.42)	0.27	0.44
	Anterior Cingulate	-0.11（-0.38-0.16）	0.42	0.49	-0.13(-0.41-0.14)	0.34	0.47
Not adjusted for global cortical amyloid level	Entorhinal Cortex	0.41（0.17-0.64）	0.001	0.01	0.37(0.13-0.60)	0.003	0.04
	Amygdala	0.29（0.07-0.52）	0.01	0.04	0.26(0.03-0.50)	0.03	0.09
	Fusiform	0.30（0.04-0.57）	0.03	0.06	0.26(-0.01-0.53)	0.05	0.12
	Parahippocampal	0.32（0.07-0.58）	0.01	0.04	0.29(0.04-0.54)	0.02	0.09
	Posterior Cingulate	0.34（0.07-0.61）	0.02	0.04	0.28(0.01-0.55)	0.04	0.11
	Occipital	0.40（0.13-0.67）	0.004	0.03	0.36(0.10-0.63)	0.01	0.05
	Orbital Frontal	0.18（-0.10-0.46）	0.21	0.28	0.07(-0.21-0.36)	0.60	0.71
	Prefrontal	0.10（-0.19-0.38）	0.49	0.54	0.06(-0.23-0.34)	0.69	0.75
	Superior Frontal	0.05（-0.23-0.33）	0.73	0.73	-0.03(-0.31-0.25)	0.84	0.85
	Lateral Temporal	0.24（-0.03-0.51）	0.08	0.14	0.21(-0.06-0.48)	0.13	0.24
	Parietal	0.22（-0.04-0.49）	0.10	0.15	0.17(-0.10-0.44)	0.22	0.31
	Posterior Precuneus	0.23（-0.05-0.50）	0.10	0.15	0.19(-0.09-0.46)	0.18	0.29
	Anterior Cingulate	-0.10（-0.36-0.17）	0.48	0.54	-0.11(-0.39-0.16)	0.42	0.54

[a] β value is coefficient of ApoE ε4 by sex interaction, 95%CI represents the 95% confidence interval of the ApoE ε4 by sex coefficient.

[b] *P* value as defined using a generalized linear model to detect significant ApoE ε4 by sex interaction effect in MCI subjects. Age, education, and MMSE score were included as covariates. Global cortical amyloid SUVR was also included as a covariate in the upper results.

[c] Adjusted *P* value as defined using Benjamini–Hochberg procedure to control FDR.

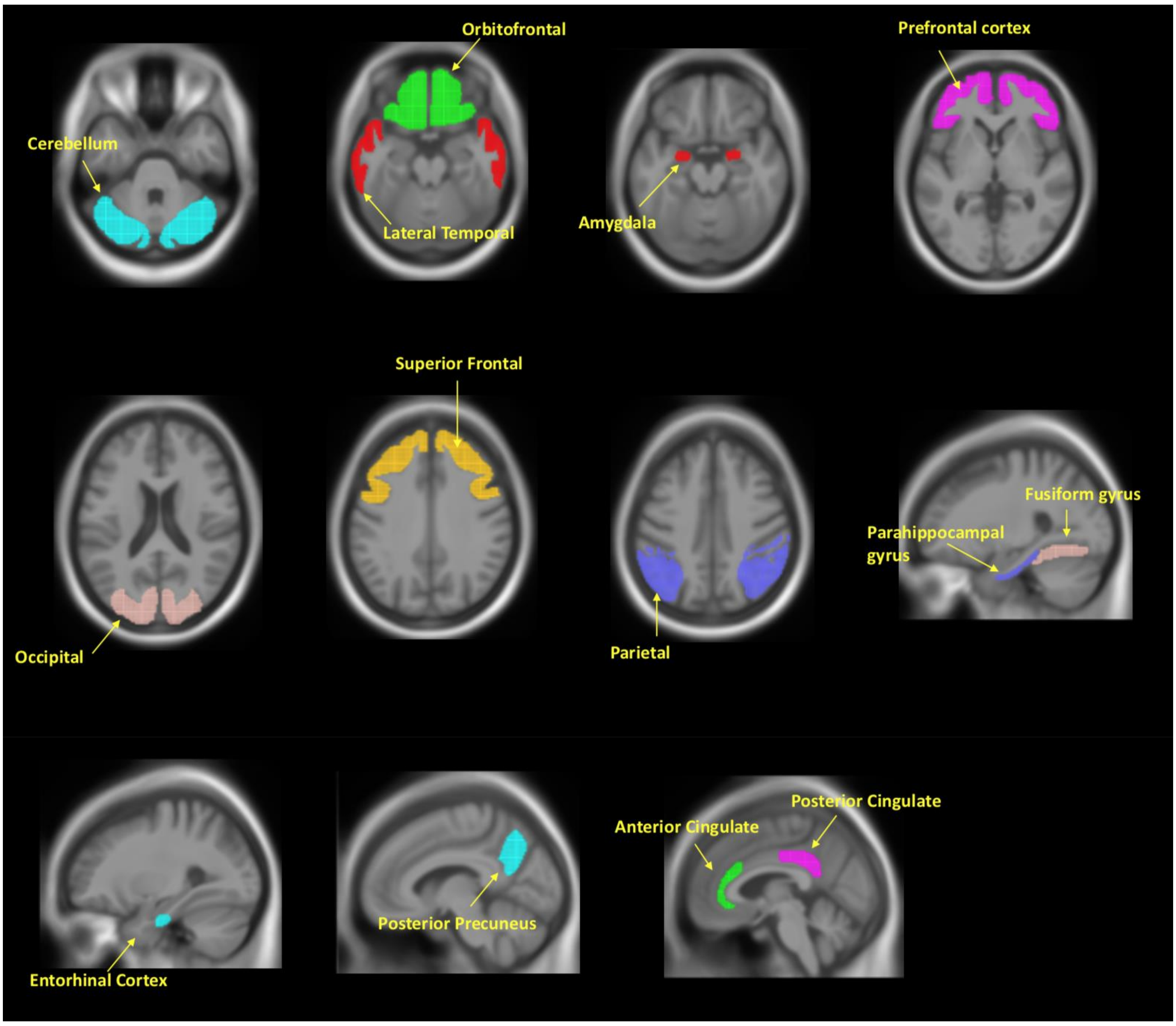

Figure S1: Regions of interest defined in standard Montreal Neurological Institute space

The ROIs used in the present study are displayed superimposed on the standard MNI MRI template. Cerebellum was used as a reference tissue. ROI SUVRs were obtained by applying the above ROIs to SUVR images in the MNI space. These 13 ROIs were selected based on our previous work in identifying cortical and subcortical regions that vary in ^{18}F-AV-1451 signal between healthy controls, MCI, and AD patients (see Methods).
